# Proton Conduction in Grain-Boundary-Free Oxygen-Deficient BaFeO_2.5+δ_ Thin Films

**DOI:** 10.3390/ma11010052

**Published:** 2017-12-29

**Authors:** Alexander Benes, Alan Molinari, Ralf Witte, Robert Kruk, Joachim Brötz, Reda Chellali, Horst Hahn, Oliver Clemens

**Affiliations:** 1 Fachgebiet Gemeinschaftslabor Nanomaterialien, Institut für Materialwissenschaft, Technische Universität Darmstadt, Alarich-Weiss-Straße 2, 64287 Darmstadt, Germany; abenes@nano.tu-darmstadt.de (A.B.); horst.hahn@kit.edu (H.H.); 2 Karlsruher Institut für Technologie, Institut für Nanotechnologie, Hermann-von-Helmholtz-Platz 1, 76344 Eggenstein-Leopoldshafen, Germany; alan.molinari@partner.kit.edu (A.M.); ralf.witte@kit.edu (R.W.); robert.kruk@kit.edu (R.K.); mohammed.chellali@kit.edu (R.C.); 3 Fachgebiet Strukturforschung, Institut für Materialwissenschaft, Technische Universität Darmstadt, Alarich-Weiss-Straße 2, 64287 Darmstadt, Germany; broetz@st.tu-darmstadt.de; 4 Fachgebiet Materialdesign durch Synthese, Institut für Materialwissenschaft, Technische Universität Darmstadt, Alarich-Weiss-Straße 2, 64287 Darmstadt, Germany

**Keywords:** pulsed laser deposition, functional thin films, electrochemistry, electrode catalysts, barium ferrite, solid oxide fuel cells

## Abstract

Reduction of the operating temperature to an intermediate temperature range between 350 °C and 600 °C is a necessity for Solid Oxide Fuel/Electrolysis Cells (SOFC/SOECs). In this respect the application of proton-conducting oxides has become a broad area of research. Materials that can conduct protons and electrons at the same time, to be used as electrode catalysts on the air electrode, are especially rare. In this article we report on the proton conduction in expitaxially grown BaFeO_2.5+δ_ (BFO) thin films deposited by pulsed laser deposition on Nb:SrTiO_3_ substrates. By using Electrochemical Impedance Spectroscopy (EIS) measurements under different wet and dry atmospheres, the bulk proton conductivity of BFO (between 200 °C and 300 °C) could be estimated for the first time (3.6 × 10^−6^ S cm^−1^ at 300 °C). The influence of oxidizing measurement atmosphere and hydration revealed a strong dependence of the conductivity, most notably at temperatures above 300 °C, which is in good agreement with the hydration behavior of BaFeO_2.5_ reported previously.

## 1. Introduction

Due to their high conversion efficiency and fuel flexibility Solid Oxide Fuel Cells (SOFCs) and their reversely operated counterparts Solid Oxide Electrolysis Cells (SOECs) are promising candidates within the fuel cell family for producing electrical energy and hydrogen fuel, respectively [1,2]. These devices are usually operated at temperatures between 800 °C and 1000 °C, leading to several high-temperature-related problems, such as high costs, fast material degradation and long start-up/shut-down times. For this reason, one of the main goals in SOFC research is to decrease the operating temperature to an intermediate temperature regime below 600 °C. The two main constraints limiting device operation only to high temperatures are the insufficient ionic conductivity of the electrolyte at lower temperatures and polarization losses, occurring at the air electrode (cathode in fuel cell mode) [3]. There are different approaches about how to tackle these problems. Minimizing the ohmic losses in the electrolyte can be realized by decreasing its thickness or by employing new material systems with higher ionic conductivity. In this respect proton-conducting electrolytes such as doped BaCeO_3_ or BaZrO_3_ have become a prominent area of research [4]. Proton migration pathways are most often characterized by lower activation barriers within solid oxides as compared to oxygen ions (protons possess smaller mass, lower radius, have no electron cloud and their transport behavior can be roughly described via a Grotthuss-related mechanism [5]) and therefore proton-conducting materials can in principle show increased ionic conductivity especially at intermediate temperatures [1,6]. Additionally, devices, employing proton conductors have attracted attention due to potentially higher electrical efficiency as has been found before [7,8,9]. A limiting factor for the application of these electrolytes is the lack of suitable electrode catalysts, which are conductive for both electrons and protons. As opposed to oxygen ion-conducting devices, the rate-limiting steps at the air electrode for proton-conducting devices depend on the proton transfer and subsequent proton migration to the electrolyte [2]. There are very few reports on a single-phase air electrode materials enabling both adequate protonic (H^+^) and good electronic (e^−^) conductivity [10,11]. In principle, a single-phase material offering simultaneous conduction of all three charge carriers (O^2−^, H^+^ and e^−^), which are involved in the electrochemical processes occurring at the air electrode, can even further enhance the catalytic activity (towards the oxygen reduction reaction in SOFC mode) as has been shown in recent reports [12,13]. Since to date there are only a few of such materials known and the determination of the individual conductivities in combination with the correct temperature range remains a scientific challenge, more research is needed. However, most of the time, one ionic charge carrier dominates the ionic conductivity: at low temperatures protons can dominate due to their higher mobility, but at higher temperatures (>600 °C) protonic transport is usually suppressed since protons cannot be stabilized within the lattice.

In the presence of oxygen vacancies water incorporation can take place in oxide materials leading to charge carrier (proton) loading, according to the following reaction in quasi-chemical Kröger-Vink nomenclature

(1) $$H_{2}O\left(g\right)+V_{O}^{••}+O_{O}^{x}\overset{\;}{\Leftrightarrow }2OH_{O}^{•}$$

where $V_{O}^{••}$, $O_{O}^{x}$ and $OH_{O}^{•}$ describe an oxygen vacancy (which is effectively 2-fold positively charged), an oxygen ion (with zero net charge) and a hydroxyl ion (with effective single positive charge) on an oxygen lattice site, respectively. The fact that oxygen vacancies enable water incorporation suggests anion deficient perovskite-type structures as possible candidates. Oxides in the perovskite-type structure are known to possess a variable oxygen stoichiometry, which may be influenced by introducing charge compensating defects in the form of aliovalent B-site substitutions such as e.g., Y in BaZr_1−x_Y_x_O_3−x/2_ [14]. Alternatively reducible B-site cations can form vacancy-ordered superstructures on the anion lattice (e.g., $BaFe_{1-x}^{IV}Fe_{x}^{III}O_{3-x/2}$) by heat treatment in reducing/oxidizing atmospheres [15]. Pure BaFeO_2.5_ (BFO) has a low mobility of oxygen ions due to vacancy ordering resulting in a complex monoclinic superstructure [16,17]. Significant oxygen ion mobility by breaking of the ordering of anion vacancies was only found at temperatures above 900 °C (diffraction studies on singly crystals at high temperatures [17]). We recently published first reports on two new hydrated phases of BaFeO_2.5_ (BFO), BaFeO_2.33_(OH)_0.33_ and BaFeO_2.25_(OH)_0.5_, highlighting the material’s ability of incorporating significant amounts of water and therefore protons [18]. In general, when examining the transport phenomena in polycrystalline samples by Electrochemical Impedance Spectroscopy (EIS) two contributions (namely bulk and grain boundary) are of interest. Since grain boundaries can have detrimental effects on the total proton conductivity, as has been shown to be the case for similar perovskite-related oxides (e.g., BaZr_1−x_Y_x_O_3−x/2_ [19]) our investigations focus on the bulk properties of BFO and a clear distinction of both contributions is necessary. Our previously conducted study on compacted hydrated powders of BFO did not allow for a clear distinction of both effects: Attempts to study the hydration behavior of dense ceramics (minimizing grain boundary contributions by sintering) were unsuccessful since during hydration of dense sintered pellets cracking and breaking occurred due to a high volume increase [18]. Such volume increases are not only limited to BFO, but were also found for the hydration of BaInO_2.5_, which can take up even higher amounts of water under the formation of BaInO_2_(OH) [20]. These large volume increases result from additional repulsive forces of the lattice cations and the incorporated protons, causing internal expansive strain [18]. Clearly, the elimination of grain boundary influences is a challenge for materials, which show strong volume increase and internal strain on water incorporation.

To eliminate grain boundary contributions completely epitaxially grown ultrathin films (~20 nm) were deposited and the process of hydration was studied in more detail at ambient temperatures [21]. The results indicated that conductivity was prevalent due to electronic charge carriers, created by partial Fe oxidation as well as lattice strain effects, which were assumed to strongly reduce protonic contributions to the total conductivity. Therefore the determination of the bulk proton conductivity within BFO still remains an open question to date.

In this work we extend our previous investigations by studying the conductivity processes, occurring in thicker epitaxial films (250 nm) of BFO grown onto single-crystal Nb-doped SrTiO_3_ substrates, as a function of different measurement temperatures (in the 200–550 °C range) and gas atmospheres (wet and dry air/Ar). The results indicate that due to the increased film thickness the lattice strain caused by the mismatch of film and substrate is reduced (with increasing distance from the interface) and in that way provides a more relaxed structure, which resembles more to the strain condition in individual crystallites found in bulk powder of BFO. Thereby an approximation of the temperature dependent bulk proton conductivity of BFO could be measured, which was not possible within the study of thinner films (~20 nm) reported previously [21].

## 2. Experimental

### 2.1. Pulsed Laser Deposition (PLD) and Thin Film Treatment

For the deposition of BFO films pulses from a KrF excimer laser (Coherent COMPexPro 205) with a wavelength of 248 nm were focused on a BFO target material over an area of around 5 mm^2^. The target for ablation was prepared by solid state synthesis as described elsewhere [16]. During film deposition the target was rotated (30 rpm) and toggled at the same time to ensure an even ablation of the material. To set the temperature of the substrate holder a diode laser, with a wavelength of 940 nm, was used. Silver paste provided the necessary thermal contact between the sample and substrate holder. The film deposition was conducted at 700 °C with a fluence of ~2 J/cm^2^ and a repetition rate of 2 Hz, leading to a deposition rate of about 3.18 nm/min (see supplementary material). The background oxygen pressure was maintained at 0.018 mbar and the substrate-target separation was set to a distance of 40 mm. All films were deposited on electronically conductive [001]-oriented Nb-doped SrTiO_3_ (0.5 wt. % Nb, Nb:STO) substrates with dimensions of 5 × 10 × 0.5 mm (width × length × thickness). The cooling rate for all deposited films was 50 K/min, while maintaining an oxygen pressure of 0.018 mbar.

After the deposition the films (samples BFO1-BFO4) were annealed at 700 °C under flowing argon for 15 h in order to minimize the Fe^4+^ contents. To observe and compare the effects of oxidation one sample (BFO5) was heated under flowing oxygen at 500 °C for 15 h. Subsequently a hydration process was performed using a wet (water of American Chemical Society (ACS) reagent grade for ultratrace analysis) stream of Argon (samples BFO2 and BFO4) at 900 mbar and 150 °C using a setup, which was described in our previous work [21]. Table 1 provides a summary of the samples, treatments and EIS measurement conditions, which were examined in this report.

### 2.2. Structural Characterization

X-ray Diffraction (XRD) measurements (θ–2θ geometry) were performed using a Rigaku SmartLab X-ray diffractometer employing Cu K_α_ radiation and a Ge 220 2-bounce monochromator on the primary side operating in reflection parallel beam geometry (170 mA, 45 kV). Some XRD measurements were performed using a Bruker D8 diffractometer (Cu K_α_ radiation) in a high-resolution setup, with 40 mA and 40 kV. The setup consisted of a Göbel mirror and a 4-bounce Ge [022] monochromator in parallel beam geometry. The ɸ-scans (‘in-plane’ structure) were recorded with a STOE 4 circle diffractometer using a Cu anode and a 2-dimensional X-ray mirror on the primary side. On the secondary side a slit system, a Ni filter and an energy dispersive detector were used to separate the Cu K_α_ line (40 mA, 35 kV).

### 2.3. X-ray Photoelectron Spectroscopy (XPS)

The surface composition and oxidation states were examined by ex-situ XPS analysis using a Physical Electronic VersaProbe XPS unit (PHI 5000 spectrometer) with Al K_α_ radiation (1486.6 eV). All detail spectra were recorded with 50.6 W, a step size of 0.1 eV and a pass energy of 23.5 eV. Whenever not specifically mentioned, the binding energies were calibrated with respect to the carbon 1s (C *1s*) emission line at 284.8 eV. To determine the integral intensity and the exact binding energies of the emission lines, the spectra in Figure 7 were background-corrected according to Shirley [22]. The atomic sensitivity factor method was used to perform a chemical analysis of the surface [23]. Sensitivity factors were provided by the Physical Electronics Handbook of XPS [24]. Since no standards could be used the described method yields semiquantitive results with 10–20% accuracy.

### 2.4. Electrochemical Impedance Spectroscopy

**Figure 1:**
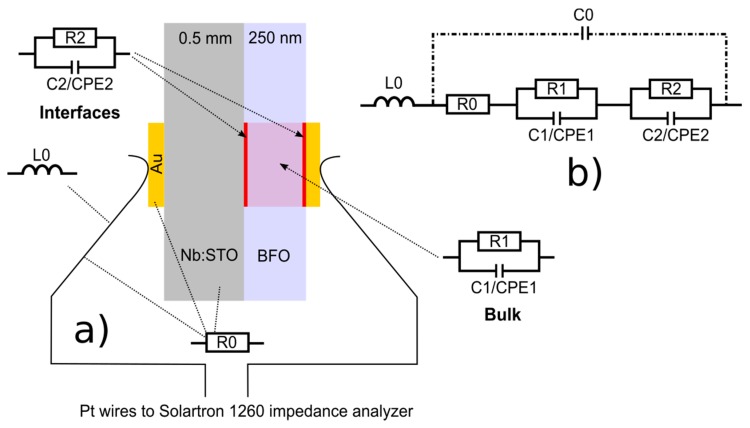
(**a**) Sample geometry for EIS measurements of BFO thin films in ‘through-plane’ geometry; (**b**) equivalent circuit model used for data fitting.

Electrochemical Impedance Spectroscopy (EIS) measurements were used to analyze the conducting properties of BFO films as a function of temperature and measurement atmosphere. For this purpose circular Au electrodes, with a diameter of 5 mm, were sputtered on both sides of the samples. The samples were then clamped in between two point contacts corresponding to through-plane measurement geometry (Figure 1a). A custom build measurement setup was used to control temperature and atmosphere. The impedance data were recorded using a frequency response analyzer (Solartron 1260, AMETEK, Berwyn, PA, USA) in the range of 1 MHz to 1 Hz with an excitation AC voltage of 20 mV. All impedance data were fitted using two conventional Constant Phase Elements in parallel to resistors accounting for film (CPE1-R1) and sample-electrode interface contributions (CPE2-R2) respectively, as shown in Figure 1. An additional resistor was assumed in series, accounting for the resistance of the measurement setup given by electrodes, wires, leads and Nb:STO substrate (which is typically in the order of 1–3 Ω). The residual capacitance C0 in parallel to the other circuit elements describes the measurement setup and was measured to be at least 5 orders of magnitude lower (1.2 pF, see supplementary material) than the film capacitance for which reason it can be neglected. The impedance of the substrate was also determined within a temperature dependent study and is negligible compared to the film impedance (~2 orders of magnitudes lower). To account for residual inductance caused by the leads an inductance was added in series to the equivalent circuit fit model (Figure 1b), so that the high frequency range (100 kHz–1 MHz) could be described correctly. The impedance spectra were fitted and evaluated using the software ZView3.4 [25]. All samples were measured during heating in the temperature range of 200 °C to 550 °C in steps of 25 °C. A continuous flow of Argon/synthetic air was used during the measurement and regulated by a Mass Flow Controller. The hydrated samples were measured in the respective wet atmosphere by bubbling the gas through water at room temperature (~3% H_2_O).

### 2.5. Conversion Electron Mössbauer Spectroscopy (CEMS)

Conversion electron ^57^Fe Mössbauer spectra of Ar annealed thin films before and after hydration were measured with a custom designed proportional counter at room temperature working with a He-CH_4_ (6%) gas mixture. Due to the low ^57^Fe concentration and the presence of several magnetically split subspectra the data acquisition time was in the order of several weeks. The Mössbauer drive is operated in linear acceleration mode and the data were fitted with the WIN Normos software package (3.0, R. A. Brand, London, UK; WissEl GmbH, Starnberg, Germany). All Isomer Shifts (IS) are given relative to α-Fe at RT.

## 3. Results

### 3.1. Film Growth and Structural Analysis

**Figure 2:**
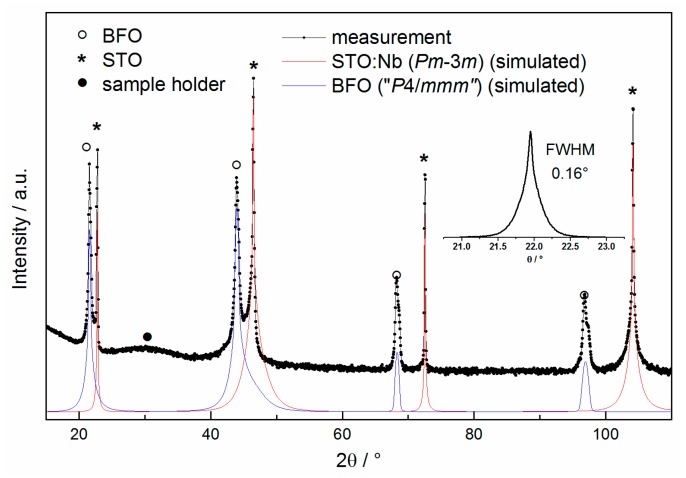
High resolution X-ray diffractogram of an as-deposited BFO film (highest possible symmetry *P*4/*mmm* due to epitaxial growth in combination with straining), showing film, substrate and sample holder reflections; the inset shows the rocking curve of the [002] reflection. Additionally, simulated intensities from structural data were added for [00l] reflections.

The crystalline structure of as-deposited, annealed and hydrated BFO films was investigated by X-ray Diffraction (XRD) analysis. BFO1 to BFO4 were also measured after EIS measurements to account for changes in lattice parameter, caused by oxidation. Figure 2 shows the θ–2θ high resolution XRD pattern of a 250 nm thick as-deposited BFO film on Nb:STO.

All films were grown epitaxially along the [00l] direction. Since the a and b lattice (in-plane) parameter is different to the c lattice (out-of-plane) parameter *c_out_*, this induces at least a symmetry lowering to a tetragonal perovskite structure (highest possible symmetry *P*4/*mmm*). Since the lattice parameters of the BFO film and the cubic perovskite substrate differ significantly, the reflections are clearly separable and can be easily identified as indicated in Figure 2. The inset in Figure 2 shows the ω rocking curve with a full-width at half-maximum (FWHM) of 0.16° for the [002] reflection of the BFO thin film. The narrow FWHM indicates a high degree of crystallinity and confirms a low mosaic spread throughout the film.

Table 2 lists the lattice parameters of the as-deposited samples and after various post-deposition treatments. The annealing treatment does not alter the out-of-plane lattice parameter *c_out_* as compared to the as-deposited film. The effect of O_2_ annealing on the other hand causes a decrease down to 3.990 Å, indicating that oxidation of Fe^3+^ to Fe^4+^ is taking place [26]. 

The ionic radius of Fe^3+^ (~64.5 pm) decreases when oxidized to Fe^4+^ (~58.5 pm) [27]. This trend is also reflected in the lattice constants respectively the cube root of the volume per formula unit (a_pseudo-cubic_) of BaFeO_2.5_ (a_pseudo-cubic_ = 4.075 Å) containing only Fe^3+^ and BaFeO_3_ (a = 3.97 Å, *Pm*-3*m*) containing only Fe^4+^ [26]. This volume decrease is well-known for bulk powders of Fe systems [28,29], for ultra-thin films and also found in the present work for 250 nm thick films of BFO. By creating mixed Fe^3+^/Fe^4+^ oxidation states an additional electronic conductivity component might be induced (small polaron hopping), in addition to reducing the concentration of the protonic charge carriers, which is why an investigation into the conducting properties, depending on atmosphere and temperature was performed and analyzed in Section 3.4 [30]. To illustrate the process of creating mixed Fe^3+^/Fe^4+^ in more detail the following equilibrium reaction in Kröger-Vink notation can be used

(2) $$V_{O}^{••}+\frac{1}{2}O_{2}+2Fe_{Fe}^{x}\overset{\;}{\Leftrightarrow }O_{O}^{x}+2Fe_{Fe}^{•}$$

where $Fe_{Fe}^{x}$ represents Fe ions in the 3+ valence state and $Fe_{Fe}^{•}$ denotes Fe ions in the 4+ valence state (similar to holes), both on Fe lattice sites. Equations (1) and (2) are in equilibrium and thereby influence each other, leading to a decrease of the proton concentration when the equilibrium in Equation (2) shifts towards the right side. In oxygen containing atmospheres this equilibrium can affect the conductivity and lead to a deviation of Arrhenius-like behavior.

**Figure 3:**
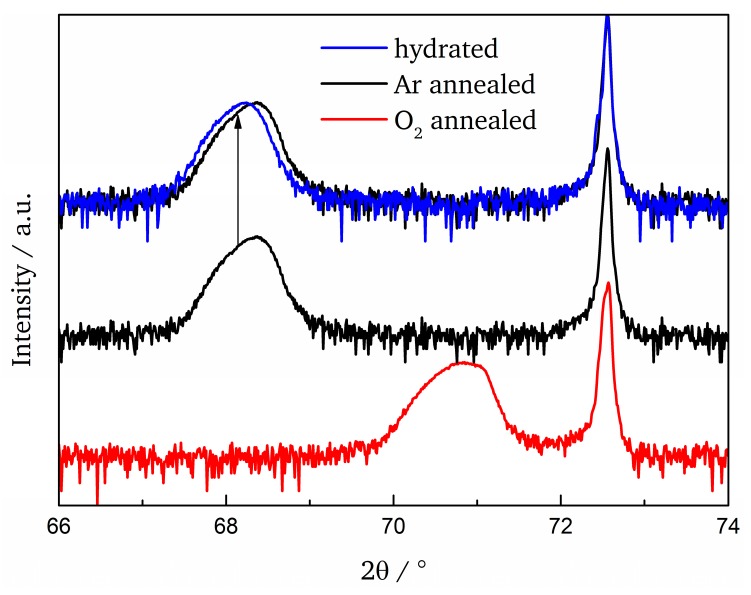
Shift of the [003] reflection due to different sample treatments. To emphasize the change in lattice parameter upon hydration the [003] reflection after Ar annealing is shifted up as indicated.

Furthermore, hydration of the film induces an expansion of *c_out_* to 4.125 Å (see Figure 5) thus proving water uptake [21]. Figure 3 displays the [003] reflection for the samples listed in Table 2. The trend of change in lattice parameter due to oxidation and hydration follows what we previously observed in ultrathin films and for bulk powders [18,21].

**Figure 4:**
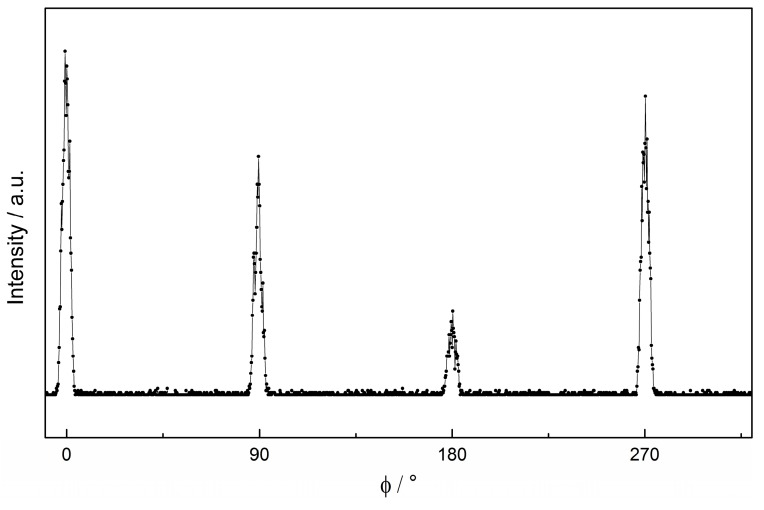
ɸ-scan of an Ar-annealed film around the [202] reflection, showing four-fold symmetry.

The ‘in-plane’ structure of the films was probed by performing ɸ-scans around the [202] asymmetric reflection. The result of a ɸ-scan (similar results for other samples) is displayed in Figure 4 confirming an epitaxial film with four-fold symmetry as is expected for a cubic (or tetragonal from symmetry lowering due to epitaxial strain) system. The reflections vary in intensity, which results from the miscut of the substrate. 

The in-plane lattice parameter *c_in-plane_* was estimated previously for ultrathin films (4.04–4.05 Å) [21]. Due to the low thickness (~20 nm) the ultrathin films were highly strained, while still being close to the pseudomorphic growth regime. The films in this study are much thicker leading to a relief of strain thus yielding a structure with properties closer to the bulk. Due to the impact of oxidation and hydration on the lattice parameter, samples BFO1 to BFO4 were measured after each EIS measurement (Table 3). While the measurement in wet Ar causes an increase in *c_out_*, the measurement in wet air slightly reduces the lattice parameter. Since oxidation and hydration have opposite effects on the lattice parameter this behavior is to be expected. Surprisingly the measurement in dry air does not show a decrease in *c_out_* to the extent, which would be expected for oxidation at elevated temperatures.

**Figure 5:**
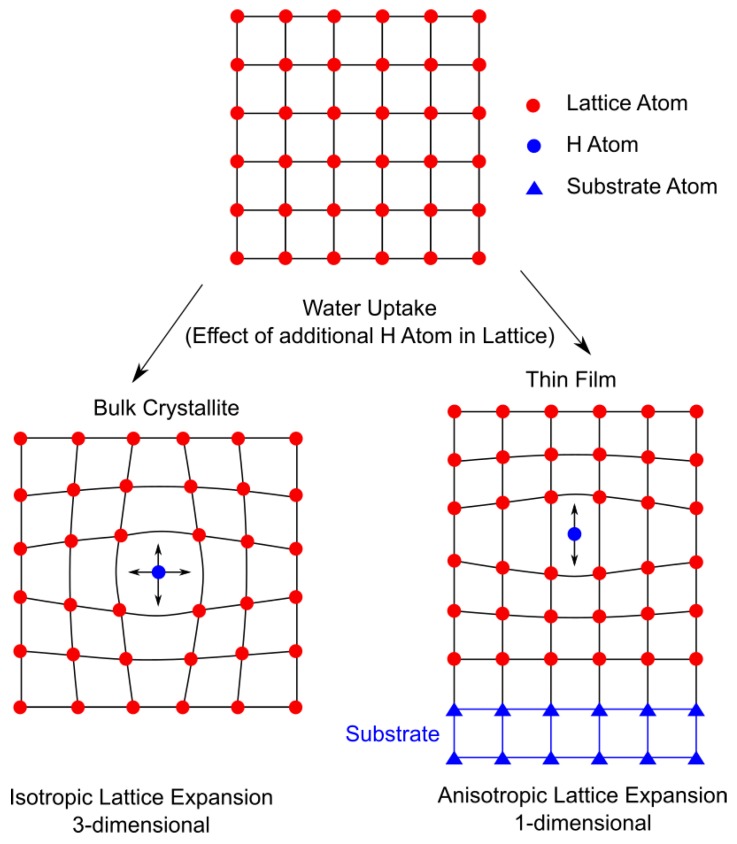
Schematic effect of proton incorporation (upon water uptake) on lattice parameter in a bulk material compared to a thin film.

From the characterization shown here we conclude that hydration and oxidation reactions show similar trends on the general change of the lattice parameter *c_out_* as compared to changes of the pseudocubic lattice parameter $\sqrt[{3}]{V_{f.u.}}$ (V_f.u._: volume per BaFeO_2.5_ formula unit) for bulk powders, although the overall magnitude still remains different [18]. Furthermore, after impedance measurements the films in air tend to show a significant oxidation, whereas the films, treated in Ar possess the same *c_out_* measured before the impedance measurement. In comparison to ultra-thin films of BFO, which were investigated previously [21], the out of plane lattice parameter of as-deposited and hydrated films is significantly shorter (4.110 Å as compared to 4.150 Å for the as-deposited and 4.125 Å as compared to 4.178 Å for the hydrated films, respectively). As mentioned above this is an indication that due to the increased film thickness the lattice strain caused by the lattice mismatch of film and substrate is reduced sufficiently so that the strain condition resembles more to the one found in individual crystallites in bulk powder of BFO. In that way it can be assumed that the films are well suited to accurately display ionic conduction in a similar manner, which would be observed in the bulk of a single crystal. However since in thin films the process of water uptake has a different effect on the lattice parameter than in bulk samples some further considerations are needed. As displayed in Figure 5, a bulk crystallite can theoretically experience isotropic expansion of the lattice in all three dimensions. For thin films the bond between film and substrate causes a lateral clamping effect, which leads to a confinement of the lattice parameter in two dimensions (in-plane). Therefore, upon water uptake of the film a lattice expansion is limited to the out-of-plane direction. Due to this anisotropic lattice expansion the water uptake and thereby the proton concentration can significantly vary in bulk samples, thin films and ultra-thin films. Table 4 lists the changes in V_f.u._ before and after hydration for different types of samples. As was already suspected due to the possibility of greater 3-dimensional lattice expansion, the bulk sample shows a significantly larger change in volume after hydration for both the low water BaFeO_2.33_(OH)_0.33_ and high water BaFeO_2.25_(OH)_0.5_ modification. Additionally, bulk samples always contain grain-boundaries and larger near surface areas, which are usually hydrated before the bulk. The ultrathin films, although showing a much lower change in V_f.u._ upon hydration than bulk samples, experience roughly double the change of V_f.u._ as compared to the 250 nm thin films deposited in this work. The difference between the 250 nm thin films and the 20 nm ultrathin films can likely be attributed to the fact that the lattice parameters differ (due to the film/substrate strain) significantly. In this way the strained ultra-thin films provide substantially more free volume for the incorporation of water. 

In summary, it can be concluded that the BFO films deposited in this work are suitable for the examination of the bulk proton conductivity (3.4), although it should be kept in mind that the proton concentration can be lower than in a bulk sample (lattice expansion and grain boundaries).

### 3.2. Local Coordination and Oxidation State of Fe

^57^Fe Mössbauer spectra were recorded to investigate the local coordination geometries and oxidation states of the iron species. With respect to the latter, the chemical shifts of all components are significant for the presence of only Fe^3+^, showing that the composition of the film can indeed be adjusted to BaFeO_2.5_ after the Ar annealing procedure [31]. Apart from oxidation states, Mössbauer Spectroscopy can yield information about the coordination geometry. Regarding the coordination geometries of the Fe polyhedra, the reader should be familiar with the following considerations:

Within a perovskite-type structure ABX_3−y_ with a cubic close packing (ccp) of the AX_3−y_ layers (i.e., which structure can be derived from the aristotype structure with space group *Pm*-*3m* (a~4 Å)), the coordination number of the anion X (here O) to the cation B (here Fe) can vary between 1 and 2, whereas the coordination number of the B cations usually varies between 4 and 6, dependent on the detailed concentration y of the anion vacancies [32]. For any fully vacancy ordered perovskite compound ABX_3−y_ where y = m/n (m, n being integer numbers, m ≤ n), the compositional formula can be rewritten as A_n_B_n_X_3n−m_. This formula can also be rewritten to indicate the local coordination schemes of the different cations and anions. To exemplify, the coordination scheme of the monoclinic bulk structure of BaFeO_2.5_ [16] is Ba_14_(FeO_4/2_)_2_(FeO_3/2_O_1/1_)_6_(FeO_5/2_)_4_(FeO_6/2_)_2_ = Ba_14_Fe_14_O_2*(4/2)+6*(3/2+1/1)+4*(5/2)+2*(6/2)_ = Ba_14_Fe_14_O_35_ = BaFeO_2.5_. Here, the sum of red numbers for each iron atom gives its coordination number, and the blue numbers are the coordination number of the respective oxygen ion to iron ions. In contrast, bulk powder of the brownmillerite-type structure of SrFeO_2.5_ [28] shows a coordination scheme of Sr_2_(FeO_6/2_)Fe(O_4/2_) = Sr_2_Fe_2_O_6/2+4/2_ = Sr_2_Fe_2_O_5_ = SrFeO_2.5_, and the coordination scheme of bulk CaMnO_2.5_ [33] is again different Ca(MnO_5/2_) = CaMnO_2.5_.

The Mössbauer signals for tetrahedrally coordinated Fe^3+^(High Spin) species are usually significantly different to the ones of octahedrally (6-fold) and square-pyramidally (5-fold) coordinated Fe^3+^(HS) species; tetrahedrally coordinated species possess lower chemical shifts as well as lower magnetic hyperfine fields as compared to the higher coordinated species (in our experience the distinction between 5- and 6-fold coordinated species from the chemical shift and magnetic hyperfine fields strongly depends on the quality of the spectra). Summing up, the Mössbauer spectra of SrFeO_2.5_ and BaFeO_2.5_ can therefore be in principle distinguished from the relative areas of the signals which correspond to 4-fold and higher coordinated Fe species (50:50 vs. ~60:40), whereas only a single signal would be expected for an Fe compound being isotypic to CaMnO_2.5_ (all Fe cations with 5-fold coordination).

**Figure 6:**
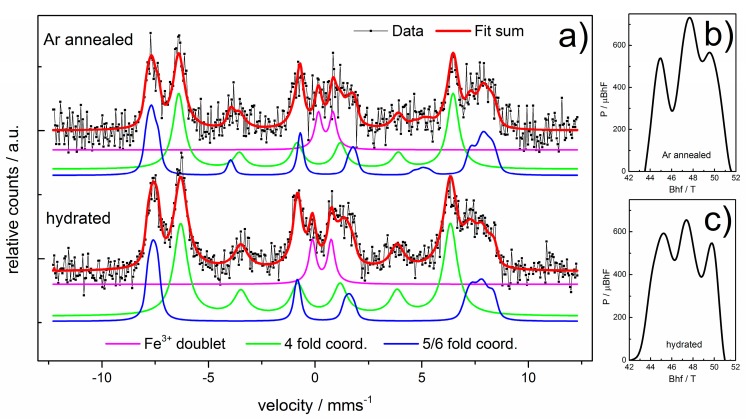
(**a**) CEMS spectra of Ar annealed samples before and after hydration and the corresponding histogram distributions of magnetic hyperfine fields for (**b**) before and (**c**) after hydration.

Although the resolution of the CEMS spectrum of the annealed BaFeO_2.5_ film (BFO1) of this study is low, it is not possible to fit the spectrum with a single sextet. Instead, at least two sextets, one belonging to 4-fold and one belonging to 5/6-fold coordinated Fe with an approximate intensity ratio of 1:1 and one doublet are needed to approximately fit the pattern. In the fit model the 4-fold coordinated sites are assigned to the well-defined sextet with the smaller hyperfine splitting (Figure 6a, green fit) of about 39 T (see supplementary material for the full set of obtained fitting parameters), in agreement with earlier results [16]. However the second very broad sextet shows a larger hyperfine field and a strong asymmetric broadening, which indicates a distribution of both hyperfine splitting and chemical shifts (Figure 6a, blue fit). It is thus reasonable to assign this subspectrum to the 5/6 fold coordinated sites. Here it is represented with a histogram distribution of magnetic hyperfine fields, which are linearly correlated with the chemical shift, in this way the number of fit parameters is minimized. Figure 6b,c present the obtained histogram distributions and the clearly observable three maxima are indicating that the local structure is similar to the case of BaFeO_2.5_ [16]. In this respect straining along [001] appears to be prohibitive for the adoption of an overall SrFeO_2.5_/BaFeO_2.5_ structural arrangement. To investigate the influence of water uptake on the oxidation state of Fe the CEMS spectrum of a hydrated sample (BFO2) was recorded for comparison. Due to the low quality of the spectra no statement in terms of a difference curve can be made at this point. However the chemical isomer shifts and obtained hyperfine parameters indicate that the oxidation state of Fe is not substantially altered and Fe^3+^ is the only iron species found after hydration.

From both measurements we conclude that Fe is present in the 3+ oxidation state, according to the isomer shifts. The corresponding magnetic hyperfine fields indicate 4-fold and 5/6-fold coordinated Fe sites. Upon hydration small changes in the fitting parameters can be observed (see Supplementary Materials), which indicate changes in the local structure due to the uptake of water. However these changes are not further analyzed due to the quality of the signal. As indicated by studies on bulk powders, the principle ordering of vacancies of BaFeO_2.5_ is quite stable up to higher temperatures, and requires temperatures above 900 °C to result in statistical distribution of oxygen sites [17]. When such vacancy ordering is released, the Mössbauer spectra give only a single signal for the iron site [34]. The fact that more than one Fe signal can be observed within the films indicates strong localization of the oxygen ions and vacancies, and therefore a low mobility.

Furthermore, the oxidation behavior of BaFeO_2.5_ is well known, showing that the material cannot be significantly oxidized under argon atmosphere. This is in agreement with our observations comparing the heating of the films under different reaction atmospheres. Additionally, the volume changes of the film are low, which is in agreement with relatively small changes of the Mössbauer spectra, and consistent with the presence of local ordering. One additional feature of the spectra is the strong suppression of the second and the fifth line of the sextet. This indicates a spin arrangement in out-of-plane direction of the film, which is related to magnetocystalline anisotropy, similarly observed for bulk powders of monoclinic BaFeO_2.5_ where the magnetic moments are aligned along the *c*-axis [16].

### 3.3. X-ray Photoelectron Spectroscopy Analysis

**Figure 7:**
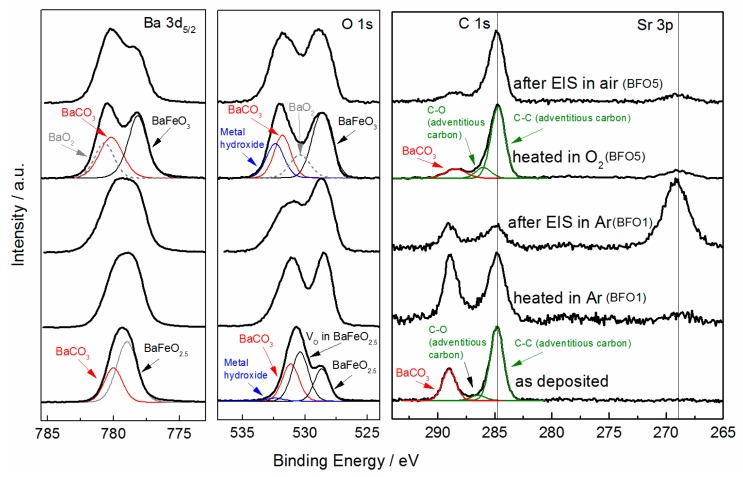
Background corrected XPS spectra of BFO thin films: Ba *3d*_5/2_, O *1s* and C *1s* (Sr *3p*).

When used as an air electrode in a solid oxide electrolysis cell the material is exposed to air at elevated temperatures. As was shown before, little amounts of oxygen can cause oxidation of Fe [21]. With Fe present in Fe^3+^/Fe^4+^ mixed oxidation states the mechanism of conduction can fundamentally change, which was shown for ultrathin films compared to bulk powders [21]. In addition Lee investigated the change in electrochemical impedance response in different atmospheres and correlated the results with the Fe^4+^ ratio contained in the bulk samples [30]. With respect to these findings the measurements in this study were conducted in oxidizing (air) and inert (Ar) atmospheres, as well as under wet and dry conditions. In order to evaluate the chemical composition of the film surfaces and oxidation states (especially of Fe) X-ray Photoelectron Spectroscopy (XPS) was used. Core level detail spectra were recorded for C *1s*, O *1s*, Ba *3d* and Fe *2p* before (after Ar/O_2_ heat treatment) and after EIS measurements. To compare the composition and changes in oxidation state the individual samples were also measured right after deposition. Figure 7 displays samples BFO1 (heated and measured in Ar) and BFO5 (heated in O_2_ and measured in air), as well as a sample after deposition. The as-deposited sample as well as the sample heated in oxygen (BFO5) are analyzed in more detail but the de-convolution of the spectra in principle also applies to the other samples, not considering the emerging Sr signal (see Supplementary Materials).

The C *1s* core level of the as-deposited sample displays three components at 284.8 eV, 286.2 eV and 288.9 eV respectively, with the first (C-C bonds) and second (C-O bonds) component being the carbon contamination due to air exposure (adventitious carbon). The third component at 288.0 eV can be attributed to carbonate groups. Correspondingly the O *1s* and Ba *3d*_3/2_ emission lines both show an additional component towards higher binding energy at 531.2 eV and at 780.0 eV, respectively. Both the Ba *3d*_3/2_ component as well as the O *1s* component have previously been associated with Ba in a non-perovskite environment, namely in BaCO_3_ [35,36,37]. In correcting the integrated peak areas by atomic sensitivity factors (ASF) the presence of BaCO_3_ is confirmed within the detectable information depth and the entailed quantitative error. Additionally, a small contribution to the O *1s* signal by metal hydroxide groups is found at 532.3 eV [35]. The components on the low binding energy side for both the O *1s* at 528.6 eV and for Ba *3d*_3/2_ at 778.9 eV are characteristic for O^2−^ and Ba^2+^ in the respective perovskite lattice. The fourth component in the O *1s* emission line at 530.3 eV is likely to arise from defects such as oxygen vacancies in the crystal structure and in that way is related to less electron-rich oxygen species [38,39].

After the deposition samples BFO1 and BFO5 were heated in Ar and O_2_, respectively. Both were then measured by EIS in the respective atmospheres (Ar/Air). The XPS measurements after both heat treatments reveal an emerging Sr *3p* emission line, which increases after the EIS measurements (clearly visible for BFO1 after EIS measurement). In this respect a minor contribution of SrCO_3_ is suspected to be present in addition to BaCO_3_ for all samples except for the as deposited. Since the only possibility of Sr on the film surface is due to diffusion/migration from the substrate, an atom probe field ion microscope was used to atomically resolve the composition across the entire film thickness (see Supplementary Materials). The incorporation of Sr throughout the film might decrease the total conductivity since e.g., SrFeO_3_ is known to not lead to hydration [40]. The implications and possible reasons for the presence of Sr throughout the film, e.g., concerning the conductivity, will be further discussed in Section 3.4.

As was already shown in Section 3.1 heating sample BFO5 in oxygen oxidizes Fe, which leads to the uptake of oxygen and thereby a drastic decrease of the oxygen vacancies in the perovskite structure. As a result the O *1s* emission line of BFO5 does not show the component, which was previously attributed to defects such as oxygen vacancies. Instead additional components on the high binding energy side of Ba *3d* (580.7 eV) and in the O *1s* spectrum at 530.4 eV are observed. These binding energies agree well with previously reported values of BaO_2_ [41] and are supported by the fact that the conditions for the formation of BaO_2_ are met during the heat treatment under oxygen (500 °C) [42]. Furthermore, the signal of the metal hydroxide groups is stronger leading to a broadening of the O *1s* emission line on the high binding energy side. For clarification the reader is informed that the adventitious carbon signal, to which the C *1s* spectra are normalized (except for BFO1 after EIS in Ar), varies to some extent depending on the sample. For this reason, the intensities of the C *1s* spectra are not easily comparable without considering the integral intensities.

**Figure 8:**
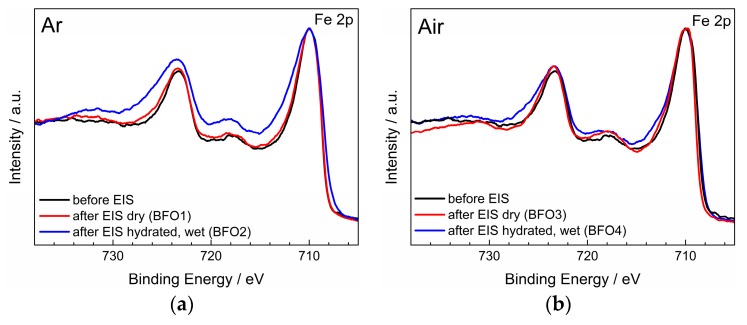
Comparison of Fe *2p* spectra after EIS measurements in (**a**) Ar and (**b**) air.

Figure 8 shows the Fe *2p* emission line for samples BFO1 and BFO2, after the EIS measurement in dry Ar and after hydration and EIS measurement in wet Ar, respectively. The measurement of an untreated sample (only Ar annealing) is added for comparison. By qualitatively comparing the shapes of the Fe *2p* emission lines, changes related to the oxidation state can be deduced. This is possible because ions of a higher positive valence state in a similar chemical environment will have a higher binding energy due to an increase in coulombic interaction of the electron cloud and the ion core [43]. For that reason the Fe *2p*_3/2_ doublets were normalized and shifted to 710 eV. The emission line of the untreated sample is sharper in comparison, indicating that before any treatment or EIS measurement the Fe exists in its trivalent oxidation state (in agreement with Mössbauer measurements reported in Section 3.2). Strong broadening of the emission lines on the high binding energy side can only be observed in the case of measurement under wet conditions (BFO2). Additionally, the satellite structure of the Fe *2p*_1/2_ peak is more pronounced. In agreement with other previous results, the change of these features suggests an oxidation of Fe and thus the presence of mixed oxidation states on the surface [44,45]. Alternatively the formation of e.g., FeOOH or Fe_2_O_3_, which would correspond to a similar change of the shape, cannot be ruled out for the hydrated samples [46]. The phenomenon of surface oxidation due to hydration was already observed previously and suggested to be caused by minor oxygen impurities [21]. Sample BFO1 on the other hand only shows very little broadening, which is to be expected for measurements conducted in inert Ar atmosphere. Figure 8b shows samples BFO3 and BFO4, which were both measured in air and consequently both show broadening on the high binding energy side, confirming a partial oxidation of Fe. In light of the probable presence of mixed Fe^3+^/Fe^4+^ valence states on the surface of BFO2, BFO3 and BFO4 an electronic contribution to the total conductivity has to be considered in the analysis of the EIS measurements (see Section 3.4).

### 3.4. Temperature Dependent Conductivity Study

**Figure 9:**
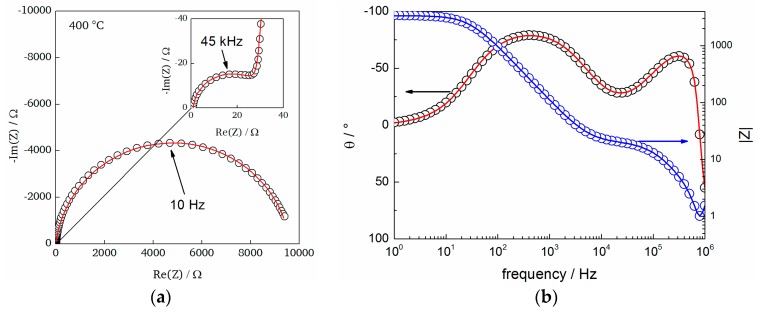
(**a**) Characteristic Nyquist plot, including Zview fits (using the model in Figure 1b) with the corresponding (**b**) bode plot for a measurement at 400 °C (Ar atmosphere).

As we examined in our previous report the protonic contribution to the overall conductivity in ultrathin BFO films (~20 nm) could not be estimated since the overall influence of the contribution of electronic charge carriers could not be determined precisely [21]. The electronic charge carriers are induced by minor oxidation reactions, which can occur on the film surface, e.g., during hydration or measurement in air. We also proposed that epitaxial strain and thereby the activation energy related to proton transport might be strongly affected in such ultrathin films. In contrast, the strain reduction in the thicker films deposited in this work makes the film more comparable to bulk BaFeO_2.5_ (also supported by the Mössbauer studies, see Section 3.2), and could therefore induce a more significant contribution of protons as charge carriers. To determine this contribution and to investigate possible effects of different gas atmospheres the films were measured at elevated temperatures in pure Ar and air, respectively. The complex-impedance plane plots of all measurements show two distinct semicircles at elevated temperatures (Figure 9). At lower temperatures (~200 °C) a single semicircle with blocking electrode behavior is observed (see supplementary material).

The semicircles were fitted using the model described in Section 2.3. The fitting parameters, which were obtained from the CPEs, were converted to effective capacitances *C_eff_* according to (3) presented by Hsu and Mansfeld [47]

(3) $$C_{eff}=Q^{1/\alpha }R_{f}^{\left(1-\alpha \right)/\alpha }$$

where *R_f_* is the resistance in parallel to the CPE and α is the constant phase element exponent related to the deviation of a straight capacitive line. In case of *α* = 1, *Q* has the units of a pure capacitor and represents the film capacitance. Both *α* and *Q* are independent of frequency. The effective capacitance values of the second semicircle were normalized to the contact area *A* confirming capacitance values for typical sample-electrode interface reactions (~8.1 × 10^−6^ F cm^−2^). The same procedure was applied to the capacitance values of the first semicircle whereas those were normalized to a geometrical factor (f = A/film thickness) and resulted in capacitance values C_eff_^*^ typical for bulk contributions (Table 5) [48]. From the resistance values of the CPE1 semicircle the conductivities were calculated and activation energies were extracted from ln(sigma*T) vs. 1/T plots.

**Figure 10:**
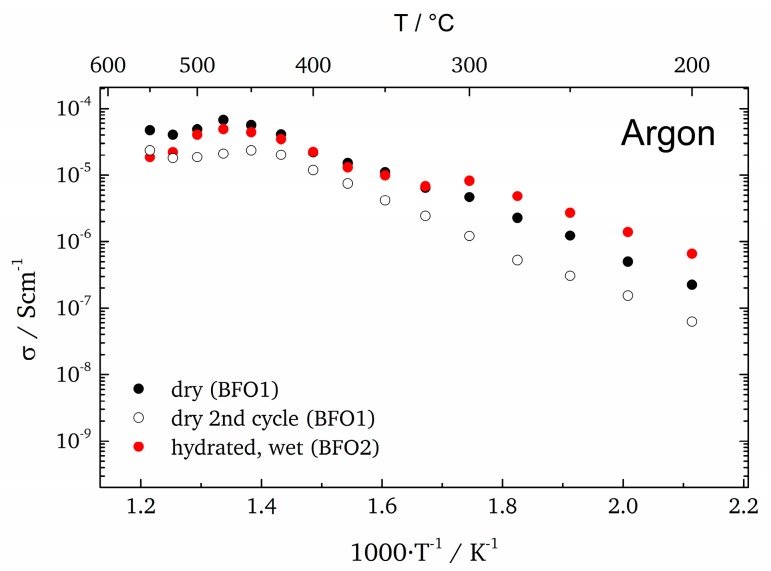
Arrhenius plots (only bulk contribution) for samples measured in dry/wet Ar during heating.

The conductivities measured in Ar atmosphere are shown in Figure 10. The measurements in dry atmospheres were conducted twice (while heating up) to track possible changes occurring in the sample due to temperature, atmosphere or applied voltage during measurement. Between 200 °C and 300 °C the hydrated sample (BFO2) exhibits increased conductivity suggesting that around 300 °C water is released from the sample. This is in agreement with the observed water loss of BaFeO_2.33_(OH)_0.33_ under transformation to BaFeO_2.5_, which coincides well with this temperature [18]. Over the remainder of the temperature range the hydrated and non-hydrated (BFO1) samples exhibit a very similar trend up to 500 °C, which is an indication that the conductivity is not dominated by a protonic contribution in this temperature range. The activation energy of 0.63 eV extracted for BFO1 in dry Ar atmosphere is comparable to the activation energy of BFO2 between 300 °C and 475 °C (EA(I) = 0.59 eV). For temperatures lower than the water release temperature, the activation energy is 0.55 eV, which is similar to what was observed for other perovskite-based proton conductors [4].

The second measurement of BFO1 shows a decrease by half an order of magnitude in conductivity as compared to the first measurement, indicating a change in structure, oxidation state or composition at the highest heating temperatures. Since neither a change in crystal structure nor a change in the oxidation state of Fe could be observed after the EIS measurements ceteris paribus, the probable cause is to be found in compositional changes. As was already discussed in Section 3.4, XPS analysis revealed Sr diffusion in connection with annealing procedures and especially EIS measurements. Gupta et al. studied Sr diffusion from STO substrates into YBa_2_Cu_3_O_7−x_ epitaxial thin films as a function of temperature [49]. Using ^85^Sr radioisotopes they traced the diffusive movement throughout the whole film at temperatures from 700 °C. Considering these results the diffusion process seems likely to be thermally activated whenever the samples are subject to high temperature (during annealing at 700 °C) or a combination of high temperature and applied voltage (during EIS measurements). During the diffusion the A-site cation, Ba^2+^ is replaced by Sr^2+^ implying that no charge compensation occurs. Furthermore, the incorporation of Sr would lead to a lattice distortion since the ionic radius of Sr (144 pm) is smaller than for Ba (161 pm) [27]. The strain induced by the lattice distortion in turn could lead to an increase of the activation energy as observed for the second EIS measurement of BFO1 (0.72 eV) as well as to a decrease in conductivity.

**Figure 11:**
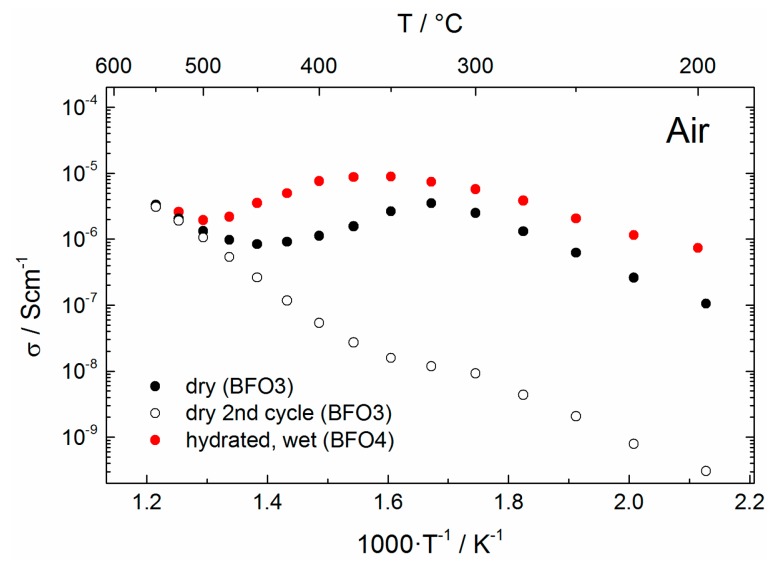
Arrhenius plots (only bulk contribution) for samples measured in dry/wet Air during heating.

Figure 11 shows the Arrhenius plots of the samples measured in air (BFO3/BFO4). As for the measurements under Ar atmosphere, the conductivity of the hydrated sample (BFO4) is significantly higher than for the sample measured in dry air (BFO3). As in the case of Ar, the conductivity of the hydrated sample (BFO4) approaches the conductivity of the dry sample (BFO3), however the conductivity values do not coincide. This is an indication that oxidative reactions take place to a different extent in wet and dry atmosphere (see Section 3.3). The hydrated sample (BFO4) shows a plateau at 350 °C, which is usually attributed to the maximum proton conductivity observed under wet conditions, proving successful hydration and indicating proton conduction [50]. The activation energy over the linear range between 200 and 300 °C is calculated assuming Arrhenius-like behavior and amounts to *E_a_* = 0.45 eV, which is typical for proton conductors [4]. Under dry conditions (BFO3) the total conductivity is lower and the activation energy in the same temperature range is higher (0.74 eV).

The higher conductivity under wet than under dry conditions coincides with the assumption that in wet atmosphere there is a significant contribution of protons to the total conductivity, in a similar way as for BFO1/BFO2 in Ar. The subsequent second measurement in dry air yields a decreased conductivity of around two orders of magnitude. The reason for this sudden decrease of conductivity can be characteristic for a proton contribution, which is lost at high temperatures above 500 °C due to dehydration or be related to effects of oxidation and/or Sr diffusion, which was previously described.

**Figure 12:**
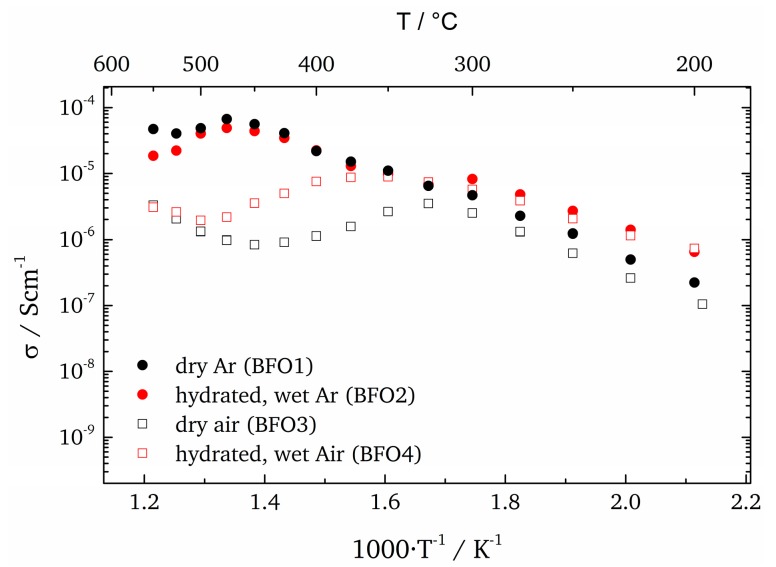
Arrhenius plots (only bulk contribution) under different measurement conditions.

Since the protonic contribution to the total conductivity is significant, a comparison of the conductivity for the samples measured under different conditions is shown in Figure 12. It is important to keep in mind that different materials release incorporated water at different temperatures and that above these temperatures the conductivity is mainly due to electronic or ionic contributions.

In general the total conductivity is given by the sum of the partial conductivities such as electronic $\sigma_{el}$ and ionic $\sigma_{ion}$ conductivity. $\sigma_{ion}$ can be divided into oxygen ionic $\sigma_{oxy}$ and protonic $\sigma_{prot}$ conductivity according to:

(4) $$\sigma_{tot}^{Ar}=\sigma_{el}^{Ar}+\sigma_{ion}^{Ar}=\sigma_{el}^{Ar}+\sigma_{oxy}^{Ar}+\sigma_{prot}^{Ar}$$

Since the difference in conductivity between wet and dry conditions (between 200 °C and 300 °C) in an inert atmosphere will mainly be due to water uptake and hydration of the film, the following relationship can be used to estimate the protonic contribution to the total conductivity:

(5) $$\sigma_{prot}\approx \sigma_{tot}^{Ar,wet}-\sigma_{tot}^{Ar,\;dry}$$

**Figure 13:**
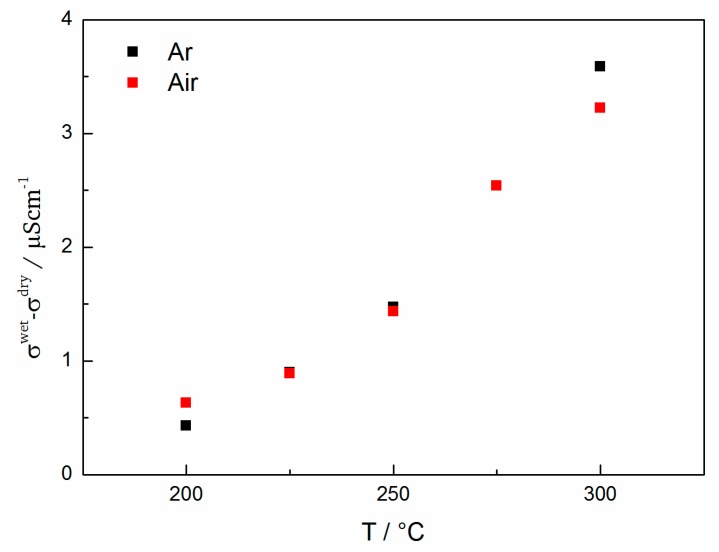
Estimation of the protonic contribution to the total conductivity for measurements in Ar and air.

In oxygen containing atmospheres this assumption can be used only in first approximation because oxidation may influence $\sigma_{oxy}$ and $\sigma_{el}$. The differences in conductivity are plotted in Figure 13 for both air and Ar atmospheres. Only temperatures between 200 °C and 300 °C are considered since water release occurs between 300 °C and 325 °C. To estimate the water release temperature the difference in conductivity of dry and wet atmospheres can be used. The temperature at which the conductivities coincide indicates that most of the water has been released from the sample.

Overall, one can conclude that there is a significant proton conductivity in BaFeO_2.5_ under wet atmospheres, although the absolute value between ~0.5 µS cm^−1^ at 200 °C to ~3.5 µS cm^−1^ at 300 °C is small compared to other known pure proton conductors such as Y-doped BaZrO_3_ [51]. However since BFO is not a pure proton conductor but can also be a good electronic conductor under certain conditions, it is rather suitable as electrode catalyst. The conductivity in most mixed ionic-electronic conductors is dominated by the electronic component (usually at least one or two magnitudes higher [52]). In BaFeO_3−δ_ the electronic component is mainly caused by p-type small polaron-hopping [52,53]. Hombo et al. correlated the Fe-O-Fe distance in their hexagonal structure of BaFeO_3−δ_ and their cubic structure of SrFeO_3−δ_ with the mobility and thereby with the total conductivity of the hopping transport [53]. Following their reasoning the samples in this work with *c_out_* ≥ 4.11 Å in a tetragonally distorted perovskite structure show longer Fe-O-Fe distances (by 0.1 Å) as compared to the ones reported, and consequently show a much lower electronic conductivity. Therefore and due to the fact that the samples in this investigation were annealed under Ar at 700 °C before EIS measurement (in that way reducing Fe^4+^ content and polaron hopping to a minimum) the electronic component and thereby the total conductivity is smaller by several orders of magnitude as compared to other literature reports. At this point, the reader should be aware that the reduction of the electronic component in this study is of crucial importance in order to allow for the determination of the protonic contribution to the total conductivity. Accordingly the protonic contribution displayed in Figure 13 constitutes a successful first determination of the bulk proton conductivity of [001]-oriented BaFeO_2.5_ in wet atmospheres. From the conclusions drawn in Section 3.1 we can furthermore estimate that the water-uptake and thereby the proton conductivity might be reduced by a factor of ~13, due to the limited volume expansion in thin films (upon hydration). When considering this factor the conductivity in this study is comparable to values, which would be expected based on earlier measurements on bulk powders [18]. For temperatures higher than 300 °C there is no discernible difference between wet and dry measurements in Ar and therefore the protonic contribution is considered to be very small. 

## 4. Conclusions

In summary, epitaxially grown [001] oriented BaFeO_2.5+δ_ films (250 nm) were structurally and electrochemically characterized in different atmospheres. Upon hydration an expansion of the out-of-plane lattice parameter of BFO was observed. Aside from examining the oxidation state and local coordination geometries Mössbauer spectroscopy also indicated changes in the local structure of Fe, which are related to uptake of water. By comparing the conductivity in wet and dry atmospheres a protonic contribution to the total conductivity could be estimated. The protonic contribution, which is present in addition to an electronic contribution stresses that the films investigated here are structurally more relaxed allowing for facilitated proton transport, as compared to ultrathin films [21]. To the best of the author’s knowledge this is the first report of bulk proton conductivity in BaFeO_2.5+δ_ thin films. The corresponding activation energies of all samples measured in wet atmospheres in the temperature range below 300 °C, coincide well with previously reported values on proton transport [4]. The temperature range above 400–500 °C (for samples measured in air) deviates from Arrhenius-like behavior due to a combination of oxidation, Sr diffusion and possibly due the influence of the EIS measurement itself. The Sr diffusion was investigated by XPS and atom probe tomography (see Supplementary Materials). Although XRD indicates that the crystal structure is not affected by this diffusion process a substitution of Ba^2+^ by Sr^2+^ in the form of Ba_1−x_Sr_x_FeO_2.5+δ_ is likely. The negative effect of Sr incorporation on the total conductivity and the process of hydration was investigated and confirmed by repeated EIS measurements. However for practical applications in SOEC devices the problem of Sr diffusion would not impose a strong problem as long as no Sr containing materials systems are utilized. In that way further investigations could focus on the study of BFO films on different substrates (e.g., BaTiO_3_) to lower A-cation interdiffusion and allow for a better understanding of film characteristics at higher temperatures.

Furthermore, it is important to correlate the findings presented here with results in our previous study on compacted powders of hydrated BFO [18]. When comparing the proton conductivities it has to be kept in mind that the water incorporation in thin films (determined from the volume expansion) is limited due to the lateral clamping caused by the substrate. Due to the fact that the conductivity σ is directly proportional to the number of charge carriers n (σ~n), the amount of water incorporated into the material must be considered when comparing the conductivities of powders to the ones of films. An extrapolation of the compacted powder conductivities determined in our earlier study results in similar values as for the hydrated thin films in this study, taking into account the limited volume expansion (σ_powder_ (200 °C, extrapolated) = 6 × 10^−5^ S/cm vs. σ_250 nm_ (200 °C) × 13 = 1 × 10^−5^ S/cm). This is an indication that the conductivity values of our previous study might have indeed been dominated by the bulk conductivity within the pellet. 

## Figures and Tables

**Table 1:** Summary of all samples including treatments and EIS measurement conditions.

Sample Name	Annealing	Hydration	EIS Measurement Conditions
BFO1	Ar	No	Dry Ar
BFO2	Ar	Yes	Wet Ar (~3% H_2_O)
BFO3	Ar	No	Dry Air
BFO4	Ar	Yes	Wet Air (~3% H_2_O)
BFO5	O_2_	No	none

**Table 2:** Out-of-plane lattice parameter after different post-deposition treatments (±0.001 Å).

Post-Deposition Treatment	*c_out_*/Å
as-deposited	4.110
Ar annealed	4.110
O2 annealed	3.990
Ar annealed and hydrated	4.125

**Table 3:** Lattice parameters *c_out_* for samples BFO1-4 after EIS measurement (±0.001 Å).

EIS Measurement Condition	Out-of-Plane Lattice Parameter (after EIS)
Dry Ar (BFO1)	4.110
Wet Ar (BFO2)	4.122
Dry Air (BFO3)	4.105
Wet Air (BFO4)	4.095

**Table 4:** Comparison of different sample types and the corresponding volumes per formula unit V_f.u._ in the dry and hydrated state. To estimate the V_f.u._ for the films in the present work the in-plane lattice parameter was assumed to be the same as for ultra-thin films (4.045 Å).

Type of Sample	V_f.u._/Å^3^	Hydrated V_f.u._/Å^3^	Change in V_f.u._ upon Hydration/Å^3^
Thin film (250 nm)	67.25	67.49	0.24
Ultra-thin film (20 nm) [21]	67.90	68.36	0.46
BaFeO_2.5_ powder (*P*2_1_/*c*) [18]	67.67	70.96 (BaFeO_2.33_(OH)_0.33_) 71.84 (BaFeO_2.25_(OH)_0.5_)	3.294.17

**Table 5:** Electrochemical parameters extracted from EIS data. Effective capacitance (C_eff_), relative permittivity (*ε_r_*), conductivity at 300 °C (*σ)*, activation energy (E_a_).

Sample (Measured in)	C_eff_ */F cm^−1^	*ε_r_*	*σ_tot_* at 300 °C/S cm^−1^	*E_a_*/eV	*E_a_*/eV 2nd Cycle
BFO1 (dry Ar)	7.8 × 10^−12^	88.1	4.7 × 10^−6^	0.63	0.73
BFO2 (wet Ar)	4.3 × 10^−12^	48.6	8.3 × 10^−6^	0.59(I)/0.55(II) *	-
BFO3 (dry Air)	1.9 × 10^−11^	214.6	2.5 × 10^−6^	0.69	0.74
BFO4 (wet Air)	1.7 × 10^−11^	192.0	5.7 × 10^−6^	0.45	-

* Two linear regimes with slightly different Ea. (I) from 200 to 300 °C and (II) above 300 °C.

## Supplementary Materials

The following are available online at www.mdpi.com/1996-1944/11/1/52/s1.

## References

[1] Fabbri E., Pergolesi D., Traversa E. (2010). Electrode materials: A challenge for the exploitation of protonic solid oxide fuel cells. Sci. Technol. Adv. Mater..

[2] Bi L., Boulfrad S., Traversa E. (2014). Steam electrolysis by solid oxide electrolysis cells (SOECs) with proton-conducting oxides. Chem. Soc. Rev..

[3] Brett D.J.L., Atkinson A., Brandon N.P., Skinner S.J. (2008). Intermediate temperature solid oxide fuel cells. Chem. Soc. Rev..

[4] Kreuer K. (1999). Aspects of the formation and mobility of protonic charge carriers and the stability of perovskite-type oxides. Solid State Ion..

[5] Münch W., Seifert G., Kreuer K.D., Maier J. (1996). A quantum molecular dynamics study of proton conduction phenomena in BaCeO_3_. Solid State Ion..

[6] Fabbri E., Bi L., Pergolesi D., Traversa E. (2012). Towards the Next Generation of Solid Oxide Fuel Cells Operating below 600 °C with Chemically Stable Proton-Conducting Electrolytes. Adv. Mater..

[7] Matsuzaki Y., Tachikawa Y., Somekawa T., Hatae T., Matsumoto H., Taniguchi S., Sasaki K. (2015). Effect of proton-conduction in electrolyte on electric efficiency of multi-stage solid oxide fuel cells. Sci. Rep..

[8] Coors W.G. (2003). Protonic ceramic fuel cells for high-efficiency operation with methane. J. Power Sources.

[9] Ni M., Leung M.K.H., Leung D.Y.C. (2007). Mathematical modelling of proton-conducting solid oxide fuel cells and comparison with oxygen-ion-conducting counterpart. Fuel Cells.

[10] Rao Y., Zhong S., He F., Wang Z., Peng R., Lu Y. (2012). Cobalt-doped BaZrO_3_: A single phase air electrode material for reversible solid oxide cells. Int. J. Hydrog. Energy.

[11] Munoz-Garcia A.B., Pavone M. (2017). K-doped Sr_2_Fe_1.5_Mo_0.5_O_6−__δ_ predicted as a bifunctional catalyst for air electrodes in proton-conducting solid oxide electrochemical cells. J. Mater. Chem. A.

[12] Kim J., Sengodan S., Kwon G., Ding D., Shin J., Liu M., Kim G. (2014). Triple-Conducting Layered Perovskites as Cathode Materials for Proton-Conducting Solid Oxide Fuel Cells. ChemSusChem.

[13] Fan L., Su P.C. (2016). Layer-structured LiNi_0.8_Co_0.2_O_2_: A new triple (H+/O_2_-/e-) conducting cathode for low temperature proton conducting solid oxide fuel cells. J. Power Sources.

[14] Kreuer K.D., Adams S., Münch W., Fuchs A., Klock U., Maier J. (2001). Proton conducting alkaline earth zirconates and titanates for high drain electrochemical applications. Solid State Ion..

[15] Johnsson M., Lemmens P. (2007). Crystallography and Chemistry of Perovskites. Handbook of Magnetism and Advanced Magnetic Materials.

[16] Clemens O., Gröting M., Witte R., Perez-Mato J.M., Loho C., Berry F.J., Kruk R., Knight K.S., Wright A.J., Hahn H. (2014). Crystallographic and magnetic structure of the perovskite-type compound BaFeO_2.5_: Unrivaled complexity in oxygen vacancy ordering. Inorg. Chem..

[17] Parras M., Gonzalezcalbet J., Valletregi M., Grenier J. (1993). A high temperature study of the BaFeO_3−y_ system. Solid State Ion..

[18] Knöchel P.L., Keenan P.J., Loho C., Reitz C., Witte R., Knight K.S., Wright A.J., Hahn H., Slater P.R., Clemens O. (2016). Synthesis, structural characterisation and proton conduction of two new hydrated phases of barium ferrite BaFeO_2.5−x_(OH)_2x_. J. Mater. Chem. A.

[19] Chen C.-T., Danel C.E., Kim S. (2011). On the origin of the blocking effect of grain-boundaries on proton transport in yttrium-doped barium zirconates. J. Mater. Chem..

[20] Fischer W. (1999). Structural transformation of the oxygen and proton conductor Ba_2_In_2_O_5_ in humid air: An in-situ X-ray powder diffraction study. Solid State Ion..

[21] Sukkurji P.A., Molinari A., Benes A., Loho C., Chakravadhanula V.S.K., Garlapati S.K., Kruk R., Clemens O. (2017). Structure and conductivity of epitaxial thin films of barium ferrite and its hydrated form BaFeO_2.5−x+δ_(OH)_2x_. J. Phys. D Appl. Phys..

[22] Shirley D.A. (1972). High-resolution X-ray photoemission spectrum of the valence bands of gold. Phys. Rev. B.

[23] Wagner C.D., Davis L.E., Zeller M.V., Taylor J.A., Raymond R.H., Gale L.H. (1981). Empirical atomic sensitivity factors for quantitative analysis by electron spectroscopy for chemical analysis. Surf. Interface Anal..

[24] Moulder J.F. (1995). Handbook of X-ray Photoelectron Spectroscopy.

[25] Johnson D. (2008). ZView: A Software Program for IES Analysis.

[26] Hayashi N., Yamamoto T., Kageyama H., Nishi M., Watanabe Y., Kawakami T., Matsushita Y., Fujimori A., Takano M. (2011). BaFeO_3_: A Ferromagnetic Iron Oxide. Angew. Chem. Int. Ed..

[27] Shannon R.D. (1976). Revised effective ionic radii and systematic studies of interatomic distances in halides and chalcogenides. Acta Crystallogr. Sect. A.

[28] Clemens O., Haberkorn R., Slater P.R., Beck H.P. (2010). Synthesis and characterisation of the Sr_x_Ba_1−x_FeO_3−y_-system and the fluorinated phases Sr_x_Ba_1−x_FeO_2_F. Solid State Sci..

[29] Clemens O., Kuhn M., Haberkorn R. (2011). Synthesis and characterization of the La_1−x_Sr_x_FeO_3−δ_ system and the fluorinated phases La_1−x_Sr_x_FeO_3−x_F_x_. J. Solid State Chem..

[30] Lee E. (2008). Characteristics of mixed conducting perovskites (Ba_1−x_Nd_x_)Fe^3+^_1−t_Fe^4+^_t_O_3−y_. J. Ind. Eng. Chem..

[31] Menil F. (1985). Systematic trends of the ^57^Fe Mössbauer isomer shifts in (FeO_n_) and (FeF_n_) polyhedra. Evidence of a new correlation between the isomer shift and the inductive effect of the competing bond *T-X* (→ Fe) (where X is O or F and T any element with a formal posit. J. Phys. Chem. Solids.

[32] Anderson M.T., Vaughey J.T., Poeppelmeier K.R. (1993). Structural Similarities among Oxygen-Deficient Perovskites. Chem. Mater..

[33] Poeppelmeier K.R., Leonowicz M.E., Longo J.M. (1982). CaMnO_2.5_ and Ca_2_MnO_3.5_: New Oxygen-Defect Perovskite-Type Oxides. J. Solid State Chem..

[34] Fournès L., Potin Y., Grenier J., Demazeau G., Pouchard M. (1987). High temperature Mössbauer spectroscopy of some SrFeO_3−y_ phases. Solid State Commun..

[35] Miot C., Husson E., Proust C., Erre R., Coutures J.P. (1997). X-ray photoelectron spectroscopy characterization of barium titanate ceramics prepared by the citric route. Residual carbon study. J. Mater. Res..

[36] Sosulnikov M.I., Teterin Y.A. (1992). X-ray photoelectron studies of Ca, Sr and Ba and their oxides and carbonates. J. Electron Spectrosc. Relat. Phenom..

[37] Christie A.B., Lee J., Sutherland I., Walls J.M. (1983). An XPS study of ion-induced compositional changes with group II and group IV compounds. Appl. Surf. Sci..

[38] García-Zaldívar O., Díaz-Castañón S., Espinosa-Beltrán F.J., Hernández-Landaverde M.A., López G., Faloh-Gandarilla J., Calderón-Piñar F. (2015). BiFeO_3_ codoping with Ba, La and Ti: Magnetic and structural studies. J. Adv. Dielectr..

[39] Fang L., Liu J., Ju S., Zheng F., Dong W., Shen M. (2010). Experimental and theoretical evidence of enhanced ferromagnetism in sonochemical synthesized BiFeO_3_ nanoparticles. Appl. Phys. Lett..

[40] Ishihara T., Bansal N.P. (2009). Perovskite Oxide for Solid Oxide Fuel Cells.

[41] Chakrabarti S., Ginnaram S., Jana S., Wu Z.-Y., Singh K., Roy A., Kumar P., Maikap S., Qiu J.-T., Cheng H.-M. (2017). Negative voltage modulated multi-level resistive switching by using a Cr/BaTiO_x_/TiN structure and quantum conductance through evidence of H_2_O_2_ sensing mechanism. Sci. Rep..

[42] Jensen W.B. (2002). Holleman-Wiberg’s Inorganic Chemistry (edited by Wiberg, Nils). J. Chem. Educ..

[43] Siegbahn C., Fahlman A., Nordberg R. (1967). ESCA: Atomic, Molecular and Solid State Structure Studied by Means of Electron Spectroscopy.

[44] Yang Y., Jiang Y., Wang Y., Sun Y. (2007). Photoinduced decomposition of BaFeO_3_ during photodegradation of methyl orange. J. Mol. Catal. A Chem..

[45] Bhargava G., Gouzman I., Chun C.M., Ramanarayanan T.A., Bernasek S.L. (2007). Characterization of the “native” surface thin film on pure polycrystalline iron: A high resolution XPS and TEM study. Appl. Surf. Sci..

[46] Grosvenor A.P., Kobe B.A., Biesinger M.C., McIntyre N.S. (2004). Investigation of multiplet splitting of Fe 2p XPS spectra and bonding in iron compounds. Surf. Interface Anal..

[47] Hsu C.H., Mansfeld F. (2001). Concernng the conversion of the constant phase element parameter Y0 into a capacitance. Corrosion.

[48] Sinclair D.C. (1995). Characterization of Electro-materials using ac Impedance Spectroscopy. Cerámica y Vidrio.

[49] Gupta D., Lacey J.A., Laibowitz R.B. (1991). Migration of Sr at the YBa_2_Cu_3_O_7−__δ_ Epitaxial Film and (100) SrTiO_3_ Substrate Interface. Defect Diffus. Forum.

[50] Bjørheim T.S., Rahman S.M.H., Eriksson S.G., Knee C.S., Haugsrud R. (2015). Hydration thermodynamics of the proton conducting oxygen-deficient perovskite series BaTi_1−x_M_x_O_3−x/2_ with M = In or Sc. Inorg. Chem..

[51] Pergolesi D., Fabbri E., D’Epifanio A., Di Bartolomeo E., Tebano A., Sanna S., Licoccia S., Balestrino G., Traversa E. (2010). High proton conduction in grain-boundary-free yttrium-doped barium zirconate films grown by pulsed laser deposition. Nat. Mater..

[52] Liu X., Zhao H., Yang J., Li Y., Chen T., Lu X., Ding W., Li F. (2011). Lattice characteristics, structure stability and oxygen permeability of BaFe_1−x_Y_x_O_3−δ_ ceramic membranes. J. Memb. Sci..

[53] Hombo J., Matsumoto Y., Kawano T. (1990). Electrical conductivities of SrFeO_3−δ_ and BaFeO_3−δ_ perovskites. J. Solid State Chem..

