# Supplementary material for: Proton Conduction in Grain-Boundary-Free Oxygen-Deficient BaFeO2.5+δ Thin Films
Source: Materials (Basel). 2017 Dec 29;11(1):52. doi: 10.3390/ma11010052 (PMC5793550; doi:10.3390/ma11010052)
Supplement: Supplementary file 1 [file materials-11-00052-s001.pdf]

materials

MDPI

Supplementary

Proton Conduction in Grain-Boundary-Free Oxygen-Deficient $BaFeO_{2.5+\delta}$ Thin Films

Alexander Benes [1,2], Alan Molinari [2], Ralf Witte [2], Robert Kruk [2], Joachim Brötz [3], Reda Chellali [2], Horst Hahn [1,2] and Oliver Clemens [1,4,*]

1 Fachgebiet Gemeinschaftslabor Nanomaterialien, Institut für Materialwissenschaft, Technische Universität Darmstadt, Alarich-Weiss-Straße 2, 64287 Darmstadt, Germany; abenes@nano.tu-darmstadt.de (A.B.); horst.hahn@kit.edu (H.H.)
2 Karlsruher Institut für Technologie, Institut für Nanotechnologie, Hermann-von-Helmholtz-Platz 1, 76344 Eggenstein-Leopoldshafen, Germany; alan.molinari@partner.kit.edu (A.M.); ralf.witte@kit.edu (R.W.); robert.kruk@kit.edu (R.K.); mohammed.chellali@kit.edu (R.C.)
3 Fachgebiet Strukturforschung, Institut für Materialwissenschaft, Technische Universität Darmstadt, Alarich-Weiss-Straße 2, 64287 Darmstadt, Germany; broetz@st.tu-darmstadt.de
4 Fachgebiet Materialdesign durch Synthese, Institut für Materialwissenschaft, Technische Universität Darmstadt, Alarich-Weiss-Straße 2, 64287 Darmstadt, Germany
* Correspondence: oliver.clemens@nano.tu-darmstadt.de

Received: 29 November 2017; Accepted: 22 December 2017; Published: 29 December 2017

Atom Probe Time of Flight (ToF) Mass Spectroscopy – Experimental section

Atom probe tomography (APT) sample preparation was performed employing a FEI Strata 400 STEM and a Zeiss Auriga 60 FIB. Prior to the lift-out, a platinum protection layer (150 nm) was deposited over the area of interest to protect the sample surface from Gallium (Ga) ion beam milling damage or sample degradation. Annular milling was used to create needle shaped morphology with an end diameter smaller than 100 nm. More details about the FIB milling process can be found elsewhere [1][2]. The APT analysis was carried out using a Cameca-LEAP 4000X HR instrument in laser pulsing mode (wave length 355 nm, pulse frequency 100 kHz, pulse energy 60 pJ, evaporation rate 0.50%) at 50 K. Data processing was achieved with the CAMECA Integrated Visualization and analysis software (IVAS- version 3.6.1), incorporating standard reconstruction algorithms, allowing the extraction of three-dimensional nanoscale chemical distribution of all detected atoms in the analysis volume.

Atom probe TOF mass spectroscopy

Due to the Sr signal detected on the surface during XPS analysis in section 3.3, atom probe tomography was used on sample BFO1 after the second EIS measurement to determine the composition as a function of growth direction. Supplementary Figure 1a shows the atomic concentrations of Ba, Fe, Sr and O plotted over the thickness of the film. No other elements, such as Ti or Nb were detected, proving that only Sr migrated/diffused from the substrate. The average atomic concentration of Sr is 10 %, which confirms the assumption of diffusion/migration throughout the complete thickness of the film. Additionally the Ba content is on average 2 % lower than the Fe content, so that as suggested in section 3.3 the replacement of Ba^{2+} by Sr^{2+} is likely to occur.

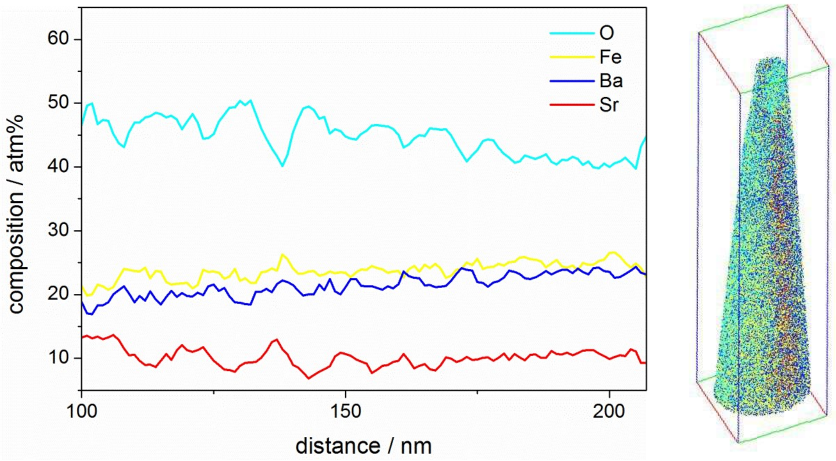

Supplementary Figure 1. a) Composition of BFO1, determined via APT analysis after second EIS measurement (in dry Ar) b) 3d atom map, 200 nm x 50 nm x 50 nm.

Mössbauer Fit Data

Supplementary Table 1. ^{57}Fe Mössbauer Spectroscopy Parameters (δ chemical isomer shift, ε effective quadrupole interaction parameter, B_{HF} magnetic hyperfine field) for $BaFeO_{2.5}$ determined from the hydrated sample in Figure 5.

Site in hydrated sample	δ / mms^{-1}	ε / mm^{-1}	BHF / T	Relative Area / %
Fe^{3+} doublet	0.42	0.88	-	9
4-fold coord.	0.20	-0.16	39.2	59
5/6 fold coord.	0.33	-0.31	47.5	32

Supplementary Table 2. ^{57}Fe Mössbauer Spectroscopy Parameters (δ chemical isomer shift, ε effective quadrupole interaction parameter, B_{HF} magnetic hyperfine field) for $BaFeO_{2.5}$ determined from the Ar-annealed sample in Figure 5.

Site in Ar annealed sample	δ / mms^{-1}	ε / mm^{-1}	BHF / T	Relative Area / %
Fe^{3+} doublet	0.60	0.68	-	9
4-fold coord.	0.21	-0.13	39.8	50
5/6 fold coord.	0.42	-0.42	48.1	41

X-ray Photoelectron Spectroscopy

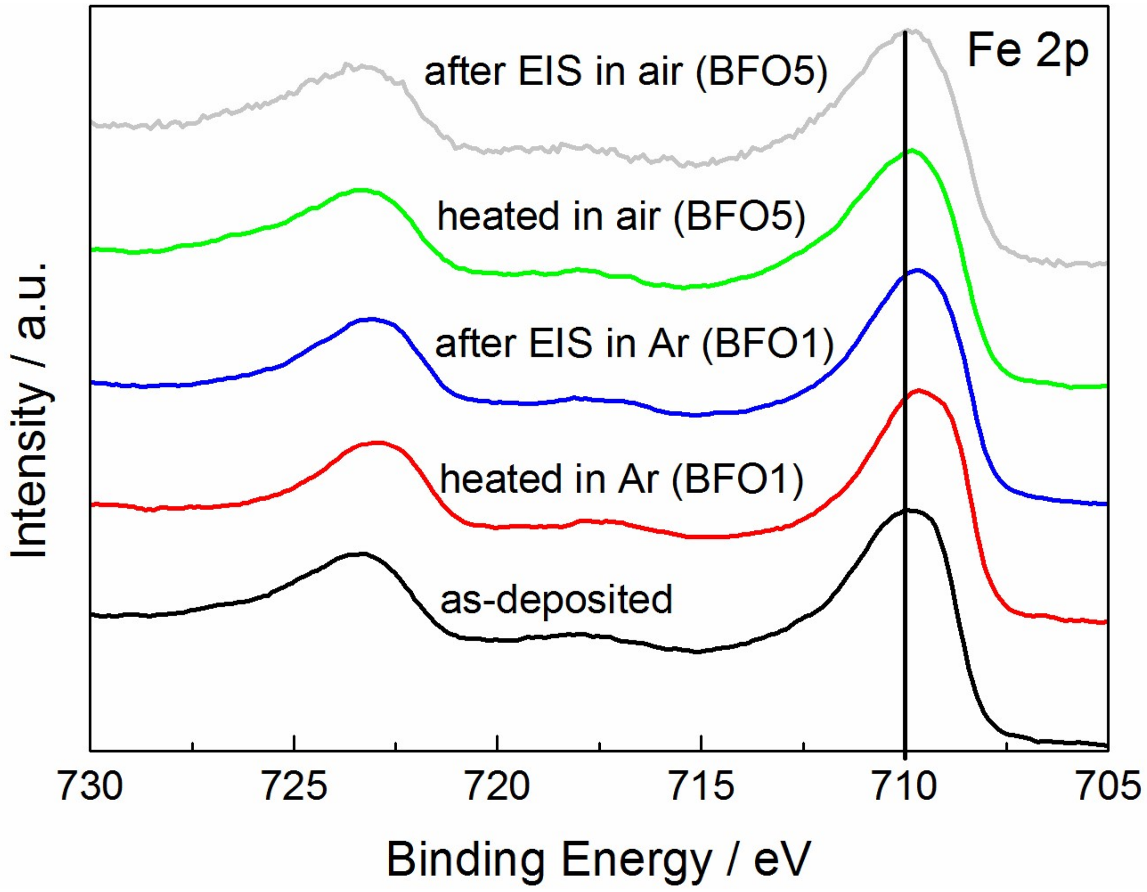

Supplementary Figure 2. Unshifted Fe *2p* XPS spectra.

Capacitance of measurement circuit

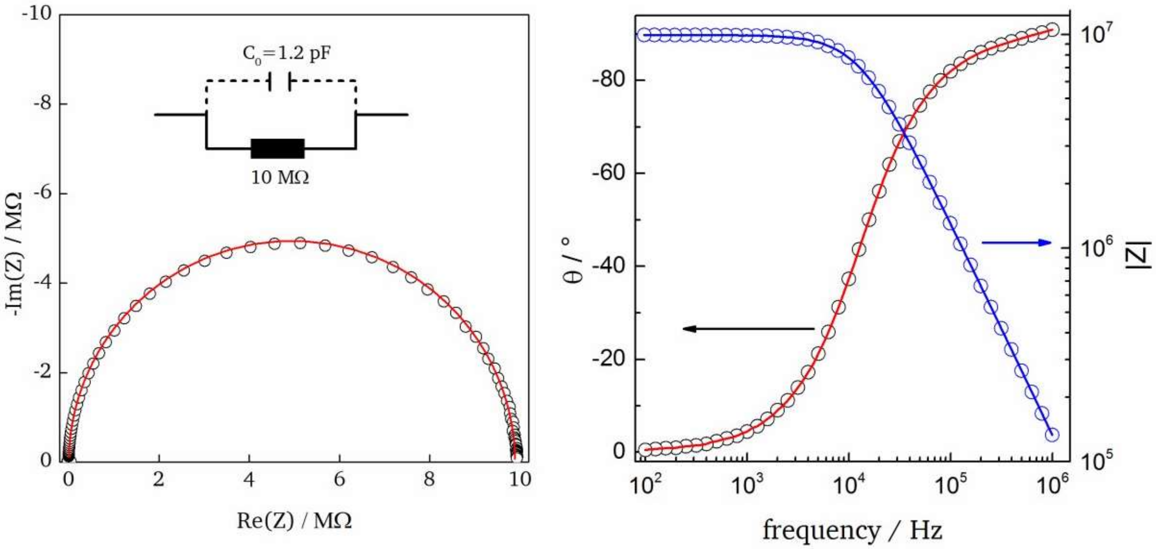

Supplementary Figure 3. Nyquist and Bode plots to determine measurement setup capacitance.

The capacitance of the measurement setup was measured by connecting a resistor (10 MΩ) in place of the substrate/film configuration. The Nyquist and Bode plots of this configuration as well as the equivalent circuit is shown in Supplementary Figure 3. The capacitance value of the setup is ~1.2

pF, which is about 5 orders of magnitude lower than the capacitances in question. Therefore this capacitance has been neglected in the calculations and equivalent circuit considerations in the article.

Additional Nyquist plots

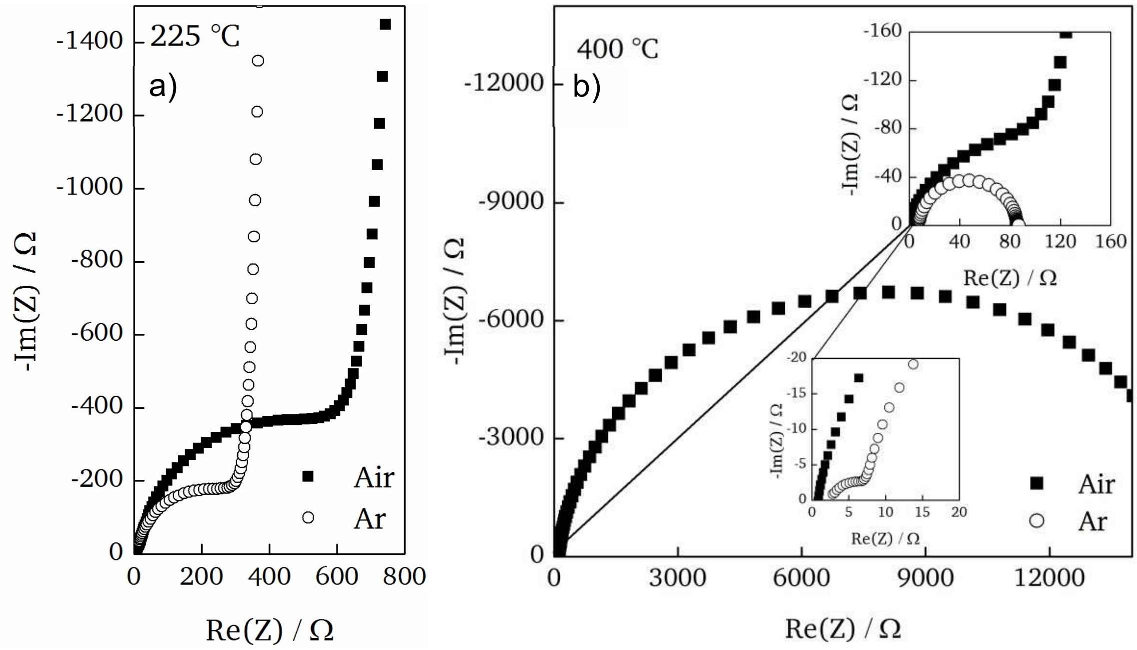

Supplementary Figure 4. Nyquist plots of measurements at a) low and b) high temperature in air and Argon.

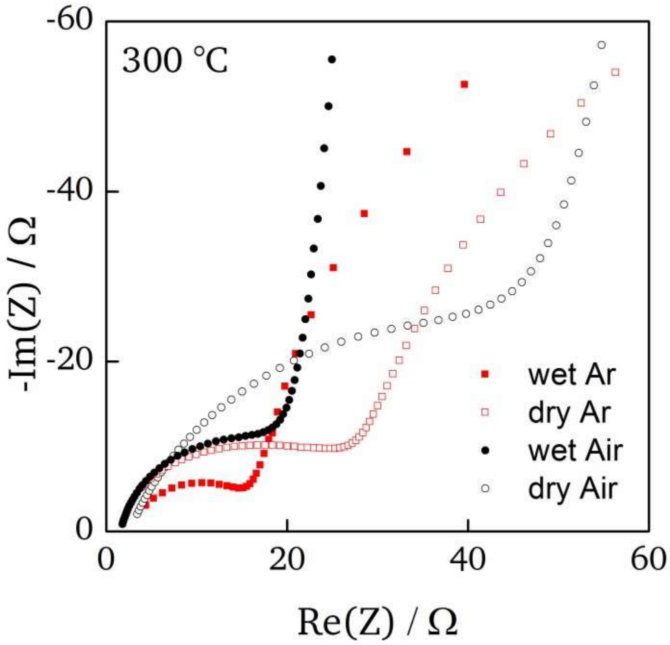

Supplementary Figure 5. Nyquist plots of measurements at 300 °C in wet/dry Ar and wet/dry air.

Substrate-only reference measurement

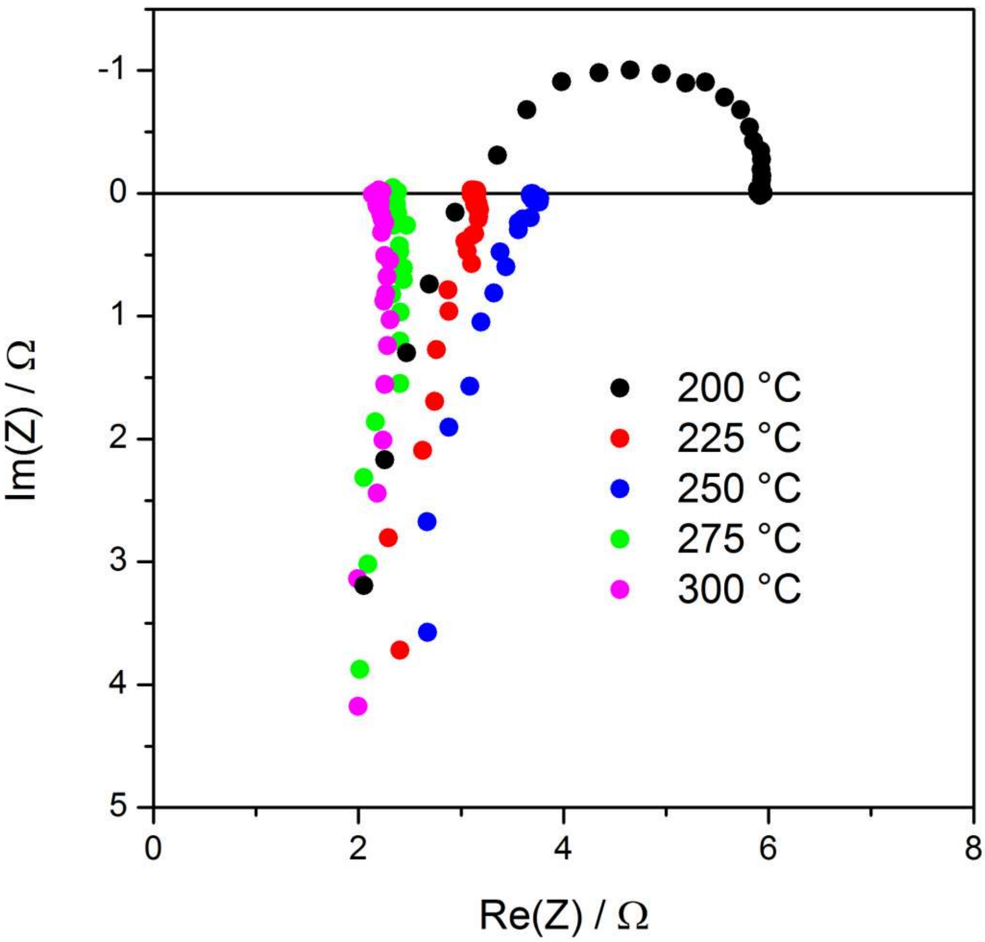

Supplementary Figure 6. Substrate only measurement in Ar.

The substrate with Au electrodes only was measured in the temperature range from 200 °C to 500 °C in Ar atmosphere. At 200 °C a substrate resistance ~ of 4 Ω could be estimated, which is small compared to the film resistance at this temperature (2 orders of magnitude larger). At temperatures higher than 200 °C the resistance of the substrate is too small to be determined. Solely the residual inductance of the leads can be observed. Supplementary Figure 6 shows the Nyquist plots of the substrate only measurements in the temperature range from 200 °C to 300 °C. The temperature range from 300 °C to 500 °C is not displayed, since it does not contain any additional information and overlaps with the measurement at 300 °C.

Film growth and microstructure

The film growth rate was estimated at 3.18 nm/min (0.53 Å/s) using X-ray reflectrometry on a Si substrate and then confirmed by Secondary Electron Microscope (SEM) micrographs. A cross-sectional and top-view micrograph of an as-deposited film are provided in Supplementary Figure 7 and Supplementary Figure 8, respectively. Right after deposition without any further treatment, the film surface presents no discernible features and the cross-section reveals homogenous film growth with a flat smooth surface in the same way as we previously observed by RHEED analysis for thinner films [3].

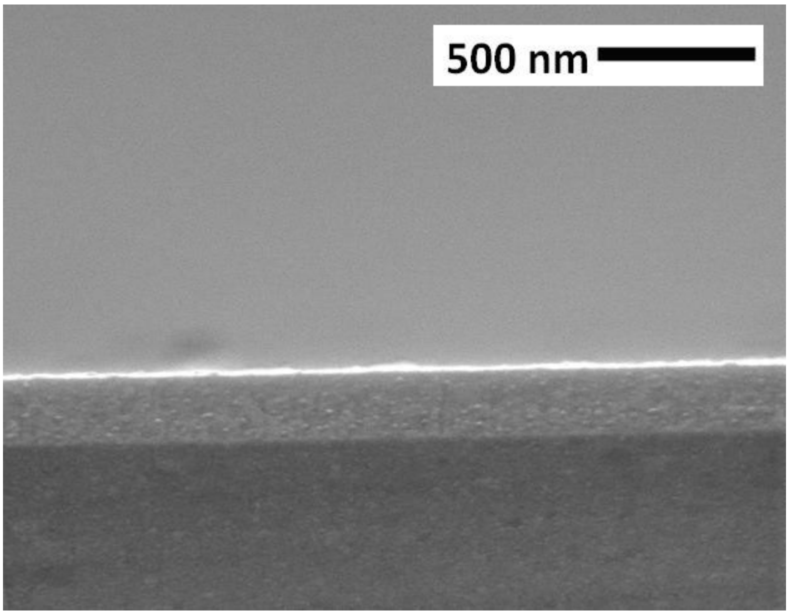

Supplementary Figure 7. Cross-sectional view of as-deposited BFO film.

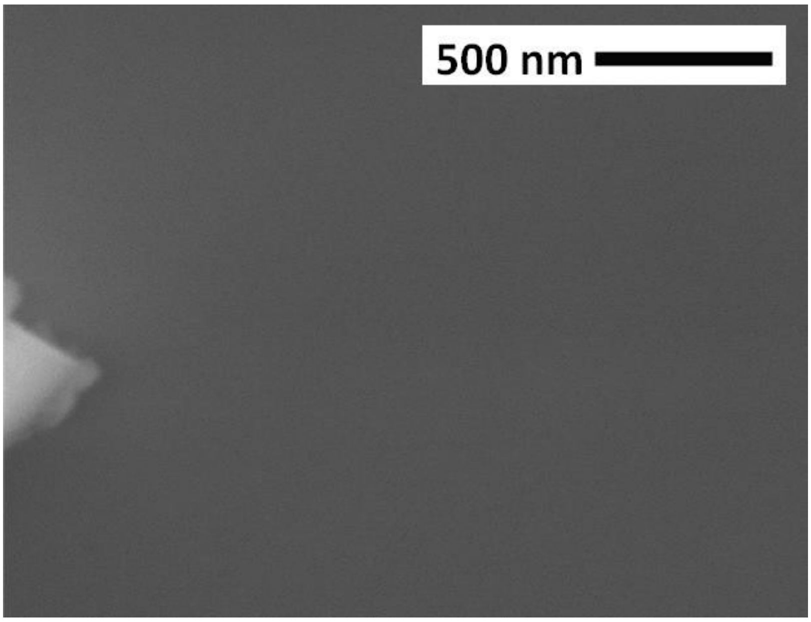

Supplementary Figure 8. Top view of as-deposited BFO film, with dirt particle used for focusing, due to lack of surface features.References.

Reference

1. Thompson, K.; Lawrence, D.; Larson, D. J.; Olson, J. D.; Kelly, T. F.; Gorman, B. In situ site-specific specimen preparation for atom probe tomography. *Ultramicroscopy* **2007**, *107*, 131–139, doi:10.1016/j.ultramic.2006.06.008.
2. Larson, D. J.; Prosa, T. J.; Ulfig, R. M.; Geiser, B. P.; Kelly, T. F. *Local Electrode Atom Probe Tomography*; Springer, 2013;
3. Sukkurji, P. A.; Molinari, A.; Benes, A.; Loho, C.; Chakravadhanula, V. S. K.; Garlapati, S. K.; Kruk, R.; Clemens, O. Structure and conductivity of epitaxial thin films of barium ferrite and its hydrated form BaFeO2.5−x+δ (OH)2x. *J. Phys. D. Appl. Phys.* **2017**, *50*, 115302, doi:10.1088/1361-6463/aa5718.

© 2017 by the authors. Submitted for possible open access publication under the terms and conditions of the Creative Commons Attribution (CC BY) license (http://creativecommons.org/licenses/by/4.0/).
